# Local infiltration of analgesia and sciatic nerve block provide similar pain relief after total knee arthroplasty

**DOI:** 10.1186/s13018-017-0616-x

**Published:** 2017-07-11

**Authors:** Hidenori Tanikawa, Kengo Harato, Ryo Ogawa, Tomoyuki Sato, Shu Kobayashi, So Nomoto, Yasuo Niki, Kazunari Okuma

**Affiliations:** 1 Department of Orthopaedic Surgery, Saiseikai Yokohamashi Tobu Hospital, 3-6-1 Shimosueyoshi, Tsurumi, Yokohama, Kanagawa Japan; 2 Department of Orthopaedic Surgery, Keio University School of Medicine, Shinjyuku, Tokyo Japan; 3 Department of Orthopaedic Surgery, Kitasato University Kitasato Institute Hospital, Minato-ku, Tokyo Japan; 4 Department of Anesthesiology, Saiseikai Yokohamashi Tobu Hospital, Yokohama, Kanagawa Japan; 5 Department of Orthopaedic Surgery, Saitama City Hospital, Saitama-shi, Saitama Japan

**Keywords:** Sciatic nerve block, Local infiltration of analgesia, Total knee arthroplasty

## Abstract

**Background:**

Although femoral nerve block provides satisfactory analgesia after total knee arthroplasty (TKA), residual posterior knee pain may decrease patient satisfaction. We conducted a randomized controlled trial to clarify the efficacy of the sciatic nerve block (SNB) and local infiltration of analgesia with steroid (LIA) regarding postoperative analgesia after TKA, when administrated in addition to femoral nerve block (FNB).

**Methods:**

Seventy-eight patients were randomly allocated to the two groups: concomitant administration of FNB and SNB or FNB and LIA. The outcome measures included post-operative pain, passive knee motion, C-reactive protein level, time to achieve rehabilitation goals, the Knee Society Score at the time of discharge, patient satisfaction level with anesthesia, length of hospital stay, surgical time, and complications related to local anesthesia.

**Results:**

The patients in group SNB showed less pain than group LIA only on postoperative hours 0 and 3. Satisfactory postoperative analgesia after TKA was also achieved with LIA combined with FNB, while averting the risks associated with SNB. The influence on progress of rehabilitation and length of hospital stay was similar for both anesthesia techniques.

**Conclusions:**

The LIA offers a potentially safer alternative to SNB as an adjunct to FNB, particularly for patients who have risk factors for sciatic nerve injury.

## Background

Postoperative pain after total knee arthroplasty (TKA) is a major concern. In an effort to reduce postoperative pain and expedite recovery, femoral nerve block (FNB) shows higher quality of postoperative status after TKA [1, 2]. Although FNB has been found to provide effective analgesia, facilitate early ambulation, and reduce the length of hospitalization in patients undergoing TKA [3–5], previous research have shown that some patients experience significant postoperative pain despite the use of FNB [6–8].

Currently, anesthetic technique of sciatic nerve block (SNB) and local infiltration of analgesic agents (LIA) are two major options to supplement FNB. Many authors have shown that LIA provide improved pain relief compared with no injection [9–12]. The analgesic effect of LIA varies due to the ingredients of the cocktail, and adding steroid in LIA has the effect of reducing inflammation, decreasing early pain relief, and improving recovery in TKA [13]. The addition of a SNB to a FNB also provides better pain relief than FNB alone [8, 14–16]. A previous study on the efficacy of SNB has shown that 67% of patients who had a preoperative FNB required the addition of a postoperative SNB [9].

The aim of this study was to compare the efficacy between SNB and LIA with steroid, when combined with the single-shot and continuous FNBs, in relieving postoperative pain, facilitating early rehabilitation, and reducing the length of hospital stay.

## Methods

### Study design

This was a double-blinded randomized controlled trial conducted in one centre. All patients received an explanation of the procedures and possible risks of the study, and gave written informed consent. This study was performed in conformity with the Declaration of Helsinki and was approved by the ethics review board at our institution. The inclusion criteria were primary TKA for osteoarthritis, American Society of Anesthesiologists (ASA) physical status classification 1–3, and full understanding of the informed consent. Exclusion criteria included patients with bilateral TKA, allergy to the drugs used in this study, neuromuscular disease, sensory disturbances of the legs, severe diabetes, heart failure, renal dysfunction, and liver dysfunction. Patients were randomized to one of two groups, combined FNB and SNB (SNB group) or combined FNB and LIA (LIA group) using sealed envelopes. Nurses and physiotherapists recording outcomes were blinded to the treatment.

### Anesthetic and surgical techniques

Following general anesthesia, the anesthesiologist conducted one-shot FNB with 20 ml of 0.375% ropivacaine and then placed a catheter tip (Aesculap, B. Braun, Melsungen, Germany) for continuous FNB. For the patients in the SNB group, the anesthesiologist conducted one-shot SNB with 20 ml of 0.375% ropivacaine using an ultrasound-guided technique in combination with a nerve stimulus technique. For the patients in the LIA group, periarticular injection of local anesthetic was undertaken by the surgeons during surgery. A solution containing 200 mg of ropivacaine (100 ml of ropivacaine 0.2%), 6.6 mg of dexamethasone, and 0.5 ml of adrenaline (1 mg · ml^−1^) was administered as follows: 20 ml administered subcutaneously around the skin incision at the beginning of surgery; 50 ml administered into the posterior capsule, collateral ligaments, and quadriceps muscles before implant fixation; 30 ml was administered into the joint space at the end of surgery. All surgeries were performed by the same team of orthopedists using three types of TKA implant model.

### Postoperative care

After surgery, 0.2% ropivacaine infusion at 5 ml · h^−1^ was initiated via the femoral nerve catheter and was continued for 48 h after surgery. Self-reported pain at rest was assessed by nurses using NRS (0 = no pain; 10 = worst pain). When a patient reported a NRS score >3 at rest, 25 mg diclofenac was administered by suppository. Nurses recorded the time when the patients were able to move his or her toes, and the following complications: nausea and vomiting, bleeding from the catheter, and toxic symptoms of local anesthesia including dizziness, tinnitus, tongue numbness, and spasm. All the patients were questioned on post-operative day (POD) 3 about the satisfaction level of the anesthesia received, which was evaluated with a categorical scale from 1 to 5 (1 = very dissatisfied, 2 = dissatisfied, 3 = neither, 4 = satisfied, 5 = very satisfied). Postoperative physiotherapy was started from the day after surgery. Maximum knee flexion angle was recorded by physiotherapists on PODs 7, 14, and 21. Physiotherapists also measured the number of days taken to achieve the following exercises: ambulation using a walker (10 m), ambulation using a cane (10 m), and climbing up and down the stairs (5 steps). Our hospital integrates acute-phase postoperative management and late-phase rehabilitation treatment, therefore the discharge criteria in our hospital includes stable 300 m ambulation using a cane, climbing up and down the stairs using a rail, and passive knee flexion of 120°.

### Outcome measures

The primary outcome measure was post-operative pain, measured on a NRS on post-operative hours 0, 3, 6, 12, 24, 48, and PODs 3 to 21. Secondary outcome measures included passive knee motion, C-reactive protein (CRP) level, time to achieve rehabilitation goals, the Knee Society Score (KSS) at the time of discharge, patient satisfaction level, length of hospital stay, induction time, surgical time, and complications related to local anesthesia. We defined the induction time and surgical time as duration from oxygen administration to initial skin incision, and duration from initial skin incision to application of surgical dressings, respectively.

### Statistics

Statistical analysis was performed using statistical software (SPSS 16.0 for Windows; SPSS Inc, Chicago, IL). The Mann–Whitney *U* test was used to analyze the nonparametric data, and the Student’s *t* test was used to analyze the parametric data. Statistical significance was defined as *P* < 0.05.

## Results

**Fig. 1:**
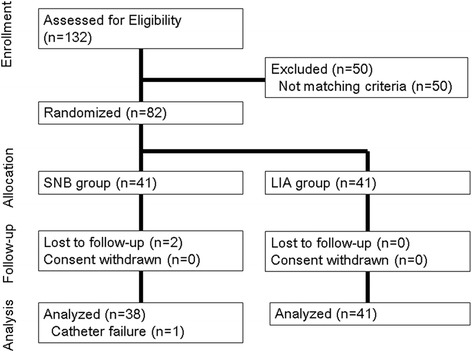
Flow diagram in line with CONSORT 2010.* indicates significant differences between LIA and SNB (*P* < 0.05). *LIA* local infiltration of analgesia, *SNB* sciatic nerve block

One hundred and thirty-two patients consented for the study. Fifty patients were excluded before randomization because they did not match the criteria. Two patients were lost to follow-up, and one patient was excluded because she removed the tube for continuous femoral nerve block by herself. Therefore, 79 patients were analyzed in this study (Fig. 1). No significant differences were observed in patient characteristics or intraoperative data between the two groups (Table 1).

**Table 1:** Patient characteristics

Variables	LIA group (*n* = 41)	SNB group (*n* = 38)	*P* value
Age (years)	74 (68–80)	76 (71–80.5)	0.356
Body mass index (kg · m^−2^)	25.0 ± 3.84	24.6 ± 3.46	0.475
Sex, F/M	30/11	29/9	0.750
ASA I/II/III	5/35/1	1/37/0	0.265

Data are presented as absolute number or median (interquartile range) as appropriate for the variables and compared by Mann-Whitney *U* test. LIA = local infiltration of analgesia; *SNB* sciatic nerve block, *ASA* American Society of Anesthesiologists

**Fig. 2:**
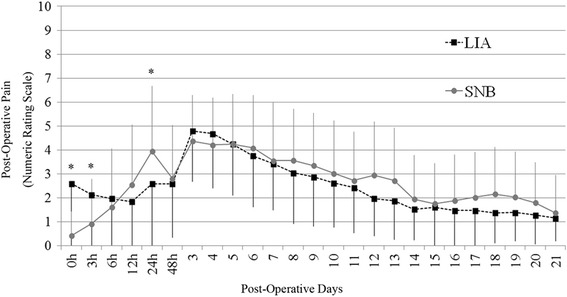
Post-operative pain (numeric rating scale) after total knee arthroplasty. Data are expressed as mean ± SD and are analyzed using the independent Student’s *t* test. * indicates significant differences between LIA and SNB (*P* < 0.05). *LIA* local infiltration of analgesia, *SNB* sciatic nerve block

The patients in group SNB showed less pain than group LIA on postoperative hours 0 and 3, and showed greater pain than group LIA on postoperative hours 24. After that, there was no significant difference between the SNB group and the LIA group concerning postoperative pain in the first 21 days (Fig. 2). The SNB group and the LIA group achieved similar passive knee flexion angle on POD 7, 14, and 21.The postoperative CRP level on POD 1 was significantly lower in LIA group compared with SNB group. The surgical time was similar between LIA group and SNB group, whereas the induction time was significantly smaller in LIA group (Table 2). There was no significant difference between the two groups in the rehabilitation progress, the length of stay, the KSS score, and the adverse effects of local anesthesia (Table 2).

**Table 2:** Post-surgical clinical data and rehabilitation milestones in LIA and SNB patients

Variables	LIA group (*n* = 41)	SNB group (*n* = 38)	*P* value
Passive knee flexion angle (degrees)			
POD 7	108 ± 13	104 ± 14	0.150
POD 14	119 ± 12	116 ± 12	0.337
POD 21	123 ± 12	123 ± 8	0.978
C-reactive protein level (mg/dl)			
POD 1	1.39 ± 0.71	3.77 ± 1.78	<0.001*
POD 3	12.14 ± 5.22	15.47 ± 4.20	0.006*
POD 7	5.53 ± 7.02	6.13 ± 8.68	0.762
POD 14	1.19 ± 1.42	1.26 ± 0.99	0.841
Time to achieve rehabilitation milestones (days)			
Ambulation with a walker	2.0 (2.0-3.0)	2.0 (1.25–3.0)	0.648
Straight leg raise	3.0 (3.0–4.0)	3.0 (3.0–4.0)	0.821
Ambulation with a cane	9.0 (7.0–11.5)	8.0 (7.0–10.0)	0.818
Stairs with a rail	11.0 (9.0–17.0)	12.0 (9.0–16.25)	0.886
Length of hospital stay (days)	24.0 (20.0–32.0)	24.0 (22.0–30.0)	0.738
Knee Society Score (function)			
Before surgery	39.2 ± 25.2	41.9 ± 28.2	0.624
At discharge	49.3 ± 16.2	51.6 ± 14.1	0.791
Knee Society Score (knee)			
Before surgery	44.9 ± 13.7	47.5 ± 17.5	0.661
At discharge	83.9 ± 10.0	84.9 ± 11.8	0.325
Induction time (min)	54.1 ± 6.4	61.0 ± 8.5	<0.001*
Surgical time (min)	73.5 ± 15.6	69.5 ± 13.1	0.215
Time of motor block in toe motion (h)	1.5 ± 4.4	6.7 ± 4.8	<0.001*
Requirement of diclofenac (mg)	0 (0–25)	0 (0–50)	0.159
Adverse effects			
Nausea or vomiting (%)	5 (12.2)	7 (18.4)	0.444
Symptoms of local anesthetic poisoning (%)	0 (0)	0 (0)	1.00

Data are presented as absolute number (percentage), median (interquartile range), or mean (SD) as appropriate for the variables and compared by Mann-Whitney *U* test or chi-square test. * Statistically significant *P* < 0.05. *LIA* local infiltration of analgesia, *SNB* sciatic nerve block, *POD* post-operative day

## Discussion

The main findings of our study show that SNB was more effective than LIA in reducing pain immediately after the surgery (within 3 h), however, SNB was less effective than LIA at 24 h after the surgery. Furthermore, 14.6% patients (six patients) in group LIA expressed severe pain (NRS > 7), whereas none of the patients in group SNB expressed severe pain. There are two possible reasons for the lower analgesic effect of LIA immediately after the surgery. First, the anesthesiologist used an ultrasound guidance technique and nerve stimulation equipment to carry out SNB, therefore accurate injection of the anesthetic agent to the sciatic nerve was achieved. In comparison, the surgeons blindly injected the anesthetic agent to the sensory nerve at the rear of knee joint in the LIA group, which may result in variability among the analgesic effect of LIA. The second reason is the timing of the procedure. The anesthesiologist performed SNB before the surgery, whereas LIA was carried out during the surgery (at the beginning of the surgery, before implant fixation, after closing the capsule), therefore the analgesic effect in LIA group might not have taken effect at the end of the surgery and at 3 h after surgery. Since the patients in LIA group expressed less pain than SNB group at 24 h after the surgery, LIA may have a longer acting time than SNB. Considering that a previous report found that 3 or less NRS score meant successful analgesia [17], our results showed that both patients in group LIA and SNB would experience enough reduction in pain after surgery. The maximum difference of NRS between the two groups was 0.98 on POD 12, which was considered to be of subclinical difference, since a change of 1.3 points on a 10 cm VAS has been reported as clinically significant [18, 19]. Our result is supported by a study comparing SNB and periarticular infiltration as an adjunct to FNB, reporting that morphine consumption, VAS scores, and knee flexion angle in the first 48 h were comparable between the two groups [16]. Although a statistically significant difference was not seen, our results show that postoperative NRS remained at a low level in both groups, and sufficient postoperative analgesia was achieved with either SNB or LIA technique combined with one-shot and continuous FNBs.

Dexamethasone is a long-acting glucocorticoid with potent anti-inflammatory properties. Its anti-inflammatory effects, both locally and systemically, were confirmed in the past study by evaluating Interleukin-6 in drain and serum CRP [13]. The postoperative CRP levels were lower in LIA group compared with SNB group, and significant difference was found in CRP levels on POD 1, possibly due to the addition of steroid to local anesthetics in the LIA group. Ikeuchi et al. evaluated the efficacy of the addition of steroid to local anesthetics in LIA and concluded that adding steroid to local anesthetics reduced inflammation, resulting in early pain relief and rapid recovery in TKA [13]. The most important possible risks with steroid in the postoperative period include gastric ulcers, impaired wound healing, and wound infections [20, 21]. These risks are mostly associated with chronic glucocorticoid use, however, careful consideration to use steroids for post-operative analgesia should be given in patients with a high-risk comorbidity prior.

The current study did not find any significant difference in progress of rehabilitation, knee mobilization, and length of hospital stay between the SNB group and the LIA group. The result makes sense given that the duration of activity of the analgesic agents were essentially limited to the first 24 to 36 h [4]. Furthermore, our result is consistent with past reports that concluded SNB or LIA were of no benefit concerning knee functional recovery or length of hospital stay [16, 22–24].

The time needed to perform SNB or LIA is also important to shorten the anesthetic time and to improve the efficiency of the operating room. The induction time was 6.9 min longer in the SNB group, while there was no significant difference in the surgical time between the two groups. LIA is an easy and fast technique, and surgeons can eliminate wasting time by injecting the drugs into the posterior of knee joint capsule while preparing the cement for implant fixation. Also, an accurate and fast SNB procedure was achieved using an ultrasound-aided peripheral nerve stimulated technique, which offers the potential benefit of accelerating the procedure, reducing the dose of local anesthetics, and resulting in higher block success rates [25–28].

SNB has similar complication rates as with any other nerve blocks, with permanent injury being exceptionally rare. Even in the absence of a SNB, TKA can place significant stress on the sciatic nerve, and sciatic nerve injury is a generally known complication after TKA with an incidence of 1.3 to 2.2% [29, 30]. Several risk factors for sciatic nerve injury after TKA have been reported, such as valgus deformity > 10°, total tourniquet time > 120 min, preexisting neuropathy, and uncontrollable postoperative bleeding [30]. Although none of the patients in SNB group sustained nerve injury in this study, it took approximately 6 h until we could confirm the toe motion. We should take account that performing a SNB could cloud the diagnosis of sciatic nerve injury and delay treatment.

There are several limitations to be noted regarding this study. Firstly, randomization by sealed envelope is open to selection bias, and a better method would have been computer generated off-site randomization to reduce this bias. Secondly, the orthopaedic surgeons who analyzed the data were not blinded to the treatment. Thirdly, the results of this study using dexamethasone cannot be applied to other types of steroid because of the variety of the pharmacological characteristics. Fourthly, combining various implant models may have an unknown effect on postoperative pain and knee function. Lastly, our hospital integrates acute-phase postoperative management and late-phase rehabilitation treatment, so the discharge criteria are different from international standard. This discrepancy may be a major restriction to expanding this study into international practice.

## Conclusions

In conclusion, satisfactory postoperative analgesia after TKA was achieved with LIA combined with FNB, while averting the risks associated with SNB, and the influence on progress of rehabilitation and length of hospital stay was similar in both anesthesia techniques. Therefore, the combination of LIA and FNB may be a safer way of anesthesia for TKA, particularly for patients who have risk factors for sciatic nerve injury after TKA.

## Abbreviations

ASA: American society of anesthesiologists
CRP: C-reactive protein
FNB: Femoral nerve block
KSS: Knee society score
LIA: Local infiltration of analgesia
NRS: Numeric rating scale
NSAIDs: Non-steroidal anti-inflammatory drugs
POD: Post-operative day
SNB: Sciatic nerve block
TKA: Total knee arthroplasty
VAS: Visual analogue scale
